# Isolation and functional verification of an aspartate aminotransferase gene from *Neoporphyra haitanensis*

**DOI:** 10.1186/s12870-023-04158-2

**Published:** 2023-03-21

**Authors:** Shuang Li, Zhanru Shao, Chang Lu, Delin Duan

**Affiliations:** 1 CAS and Shandong Province Key Laboratory of Experimental Marine Biology, Center for Ocean Mega-Science, Institute of Oceanology, Chinese Academy of Sciences, Qingdao, 266071 China; 2 Laboratory for Marine Biology and Biotechnology, Qingdao National Laboratory for Marine Science and Technology, Qingdao, 266237 China; 3 University of Chinese Academy of Sciences, Beijing, 100049 China; 4 Department of Biological Engineering, College of Life Science, Yantai University, Yantai, 264005 P. R. China

**Keywords:** *Neoporphyra haitanensis*, Aspartic acid, Aspartate aminotransferase, Transcriptional expression, Kinetic parameters

## Abstract

**Background:**

*Neoporphyra haitanensis* is a commercial laver species in China. Aspartic acid is an important flavor amino acid, and aspartate aminotransferase (AAT) is a crucial enzyme in its biosynthesis. In this study, we cloned one AAT gene (*NhAAT*) from the red alga *N. haitanensis* and investigated its sequence structure, transcriptional expression and enzymatic characteristics. The purpose of our research is to obtain a functional AAT responsible for the biosynthesis of aspartic acid from red seaweeds, which has the potential to influence the flavor of *N. haitanensis*.

**Results:**

Sequence analysis showed that NhAAT contains a conserved domain of Aminotran_1_2, which belongs to the transaminase superfamily. The secondary structure of NhAAT is dominated by α-helix. The results of enzymatic characterization illustrated that the NhAAT has highest catalytic activity at 45 °C and pH 7.5 in both forward and reverse reactions. The calculated *K*_*m*_ values of NhAAT was 5.67 and 6.16 mM for L-glutamic acid and L-aspartic acid, respectively. Quantitative analysis showed that the *NhAAT* expression of *N. haitanensis* collected in late harvest (Dec) was 4.5 times that of *N. haitanensis* collected in early harvest (Oct), while the aspartic acid content of *N. haitanensis* collected in late harvest (Dec) was 1.2 times that of *N. haitanensis* collected in early harvest (Oct).

**Conclusion:**

The results of enzyme kinetics indicated that NhAAT prefers to catalyze the reaction in the direction of aspartic acid production. Moreover, the trend of *NhAAT* expression level was consistent with that of aspartic acid content in *N. haitanensis* in different harvest periods. Our research is helpful to understand the accumulation and regulation of amino acids in *N. haitanensis* in different habitats and the taste difference of *N. haitanensis* in different harvest periods.

**Supplementary Information:**

The online version contains supplementary material available at 10.1186/s12870-023-04158-2.

## Background

Traditionally, laver is the staple food in limited areas of Asia, but its health benefits have led to a sharp increase in consumption around the world [1]. *N. haitanensis* has become an economic seaweed, which can be used in various food industries. According to China Fishery Statistical Yearbook 2021, the total output of laver in China has reached 222,018 tons, and the cultivation area has reached 72,399 hectares, of which the main economic species are *Neopyropia yezoensis* and *N. haitanensis*.

*N. haitanensis* is popular ascribing to its high nutritional value and characteristic flavor, so flavor is an important quality index for *N. haitanensis*. Flavor amino acid (FAA) mainly includes sweet amino acid (SAA) and umami amino acid (UAA) [2]. The content of FAA determines the flavor of *N. haitanensis*. Each free amino acid will produce sweet, bitter, sour or delicious characteristics for food, and aspartic acid can elicit umami taste [3]. *N. haitanensis* contains a high content of aspartic acid [2], which has an important impact on the flavor of *N. haitanensis*.

**Fig. 1:**
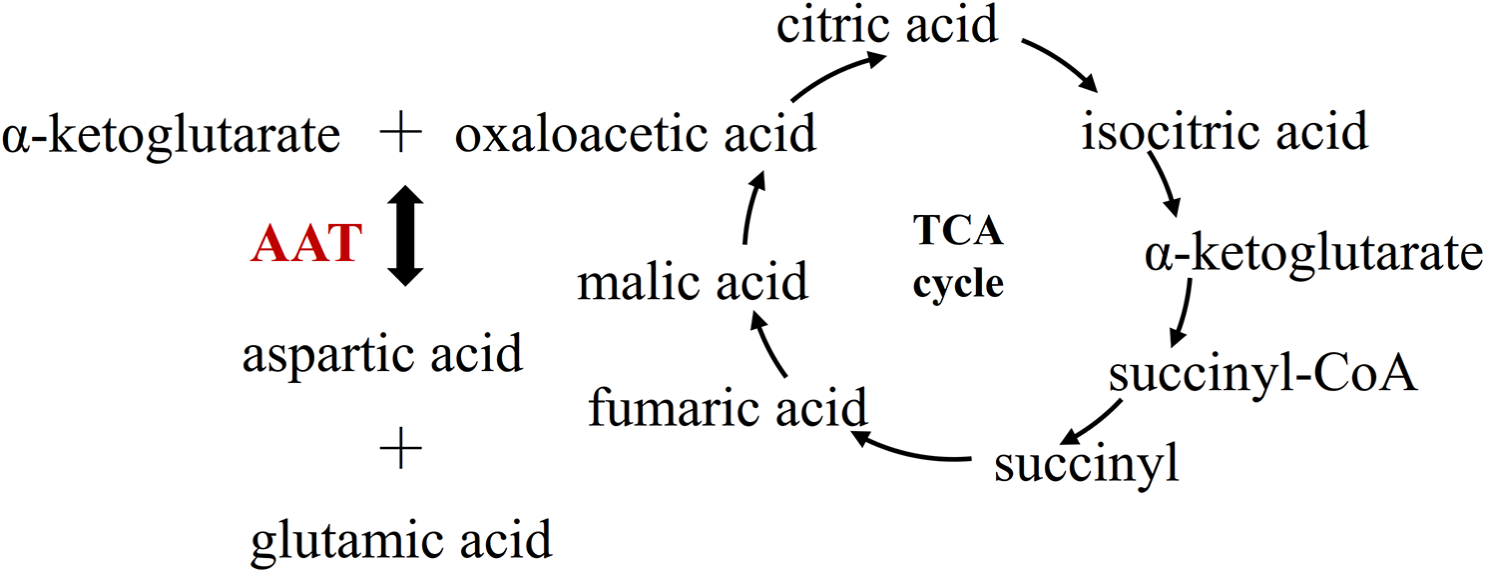
The aspartic acid biosynthetic pathway

In addition to being a flavor amino acid, aspartic acid also has many important physiological functions in vivo. It can transfer the reduction equivalent produced in glycolysis to the mitochondrial membrane for oxidative phosphorylation and produce ATP through the malic acid aspartic acid shuttle pathway [4]. The metabolism of aspartic acid involves the biosynthesis of many key substances [4]: pyrimidine [5], arginine [6], NAD [7] and so on. The biosynthesis pathway of aspartic acid in vivo was indicated in Fig. 1. The oxaloacetic acid can receive the amino group transferred from glutamic acid to produce aspartic acid. This reaction is catalyzed by aspartate aminotransferase (AAT) [4].

AAT, also known as glutamic oxaloacetic transaminase, is a typical prototypical pyridoxal 5’-phosphate (PLP) dependent enzyme. AAT belongs to transaminase superfamily I, which can catalyze the reversible reaction of oxaloacetic acid and L-glutamic acid to produce L-aspartic acid and α-ketoglutarate. The reaction process catalyzed by AAT is completed through two semi reactions in the ping pong kinetic mechanism [8]: the first step is that PLP reacts with L-glutamic acid to produce pyridox-amine 5’-phosphate (PMP) and α-ketoglutarate; in the second step, the semi reaction between PMP and oxaloacetic acid regenerates PLP and obtains the product L-aspartic acid. Although the three-dimensional structure of all AATs is conservative, there are significant differences in their primary structure [9, 10]. There are various AAT isozymes in plants located in specific subcellular compartments: cytoplasmic matrix, mitochondria, peroxisomes and plastids [11].

So far, the research of aspartic acid in algae mostly focuses on the comparison of amino acid content among different species [1, 12] and the environmental conditions affecting its content [13]. At present, the gene mining and activity analysis of AAT in *N. haitanensis* have not been explored. In this study, we cloned one AAT gene (*NhAAT*) from the red alga *N. haitanensis* and investigated its sequence structure, transcriptional expression and enzymatic characteristics. Our research is helpful to understand the accumulation and regulation of amino acids in *N. haitanensis* in different habitats and the taste difference of *N. haitanensis* in different harvest periods.

## Results

### Sequence analysis of NhAAT

The sequence features of NhAAT are summarized in Table 1. The ORF of *NhAAT* (1,290 bp) encoded 429 amino acids, which had a predicted molecular mass of 45.04 kDa and an isoelectric point (pI) of 7.11. Secondary structure prediction showed that the main secondary structure of NhAAT is α-helix structure (~ 45%).

**Table 1:** Characteristics of NhAAT in terms of gene/protein structure and subcellular localization

Name	NhAAT
Length of CDS (bp)	1,290
Size of protein (AA)	429
Molecular mass (kDa)	45.04
Isoelectric point	7.11
α-helix (%)	44.99
Extened strand (%)	13.52
β-turn (%)	6.76
Random coil (%)	34.73
Signal peptide	0
Subcellular location	OT
Transmembrane helices	0
Conserved domain	Aminotran_1_2

AA, amino acids; CDS, coding sequences; OT, other

**Fig. 2:**
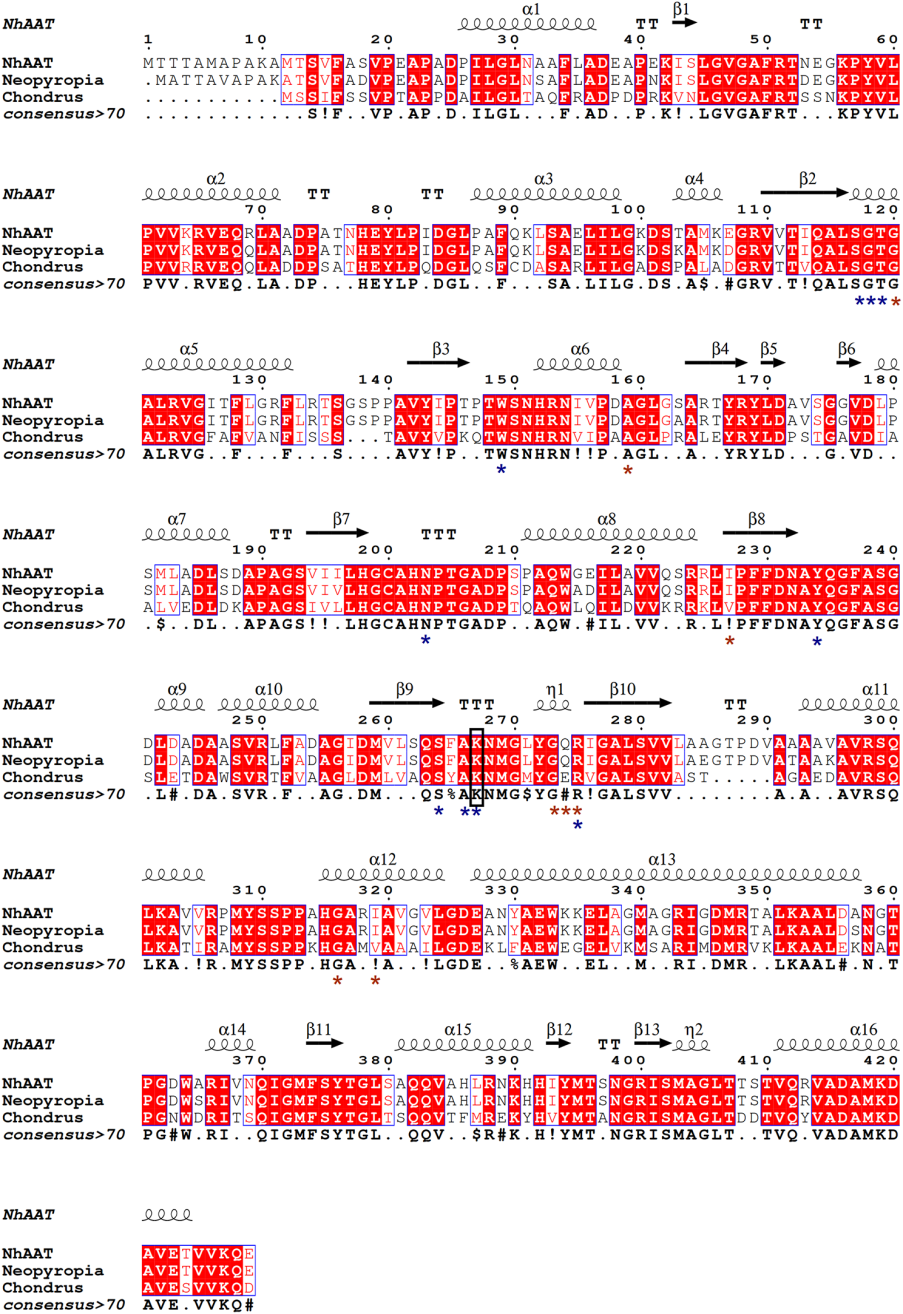
Multiple sequence alignment of NhAATs with AATs from *N. yezoensis* and *C. crispus.* The binding sites of the PLP are marked with blue asterisks, and the binding sites of the polypeptide are marked with red asterisks. The amino acid residue in black boxes is the catalytic residue

Figure 2 showed multiple sequence alignment results of NhAAT with AATs from *N. yezoensis* (NCBI accession: AIT70268.1) and *Chondrus crispus* (NCBI accession: AIT70261.1), and the predicted important sites related to cofactors binding and dimerization have been marked. According to the conserved protein domains analysis by NCBI, the amino acid residues participating in the binding of PLP (blue asterisks) and dimerization (red asterisks) were retrieved. Moreover, the Lys^267^ (in black boxes) is predicted to be a catalytic residue. From the results of multiple sequence alignment, these important sites were relatively conserved in red algal AATs.

To construct a phylogenetic tree, we selected four AATs from land plants, four from Rhodophyta, and four from Phaeophyta. And from the tree, we can see the evolutionary position of land plants is later than that of red algae (Figure S1).

### Expression and purification of NhAAT

**Fig. 3:**
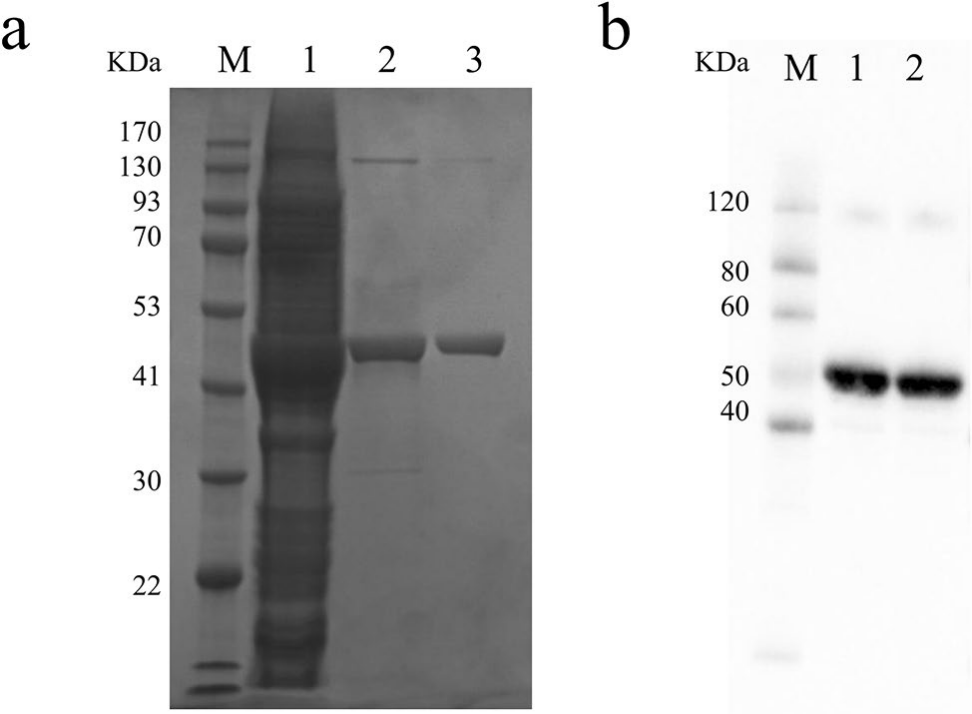
SDS-PAGE and Western blot results of recombinant NhAAT. (a) SDS-PAGE analysis of recombinant NhAAT, M: protein ladder, Lane 1: crude enzyme; Lane 2: purified NhAAT after affinity purification, Lane 3: purified NhAAT after gel filtration purification. (b) Western blot analysis of recombinant NhAAT, M: protein ladder, Lanes 1–2: purified NhAAT after gel filtration purification. The protein was detected with His-tag antibody. To improve the clarity and conciseness of the presentation, the gel and blot have been cropped. Original SDS-PAGE and Western blot results are shown in the Fig. S2 (Supplementary information)

To functionally characterize NhAAT and compare their enzyme characteristics, we first induced large amounts of NhAAT protein expression. The molecular mass of NhAAT His-tag fusion protein was about 46 kDa. Recombinant His-tagged NhAAT was purified, and the SDS-PAGE (Fig. 3a) and Western blot (Fig. 3b) analysis confirmed the presence of proteins with the expected sizes.

### Enzyme activity analysis of NhAAT

**Fig. 4:**
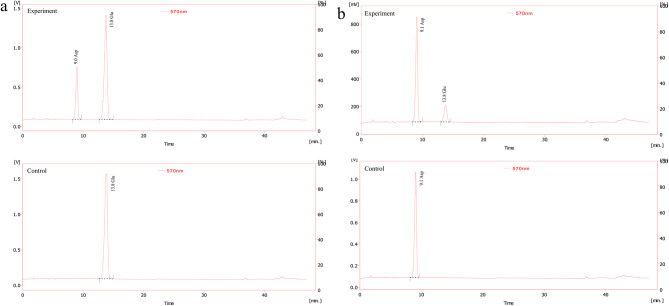
HPLC detections of the forward and reverse enzyme reaction system. (a) forward enzyme reaction system. upper panel, experimental group, substrates incubated with NhAAT; lower panel, control group, substrates incubated with inactivated (i.e., boiled) NhAAT. (b) reverse enzyme reaction system. upper panel, experimental group, substrates incubated with NhAAT; lower panel, control group, substrates incubated with inactivated (i.e., boiled) NhAAT. The reaction was carried out at 30℃ for 5 min, and then the enzyme was inactivated at 98℃ to terminate the reaction

The amino acid substrate and product of enzyme activity reaction were determined by ninhydrin post column derivation method. The enzyme reaction mixture was tested by HPLC, and the inactivated enzyme was used as control. Except for the substrate of glutamic acid, the formation of aspartic acid was detected in the products of the experimental group, while the control group had no corresponding peak (Fig. 4a). This indicates that the recombinant NhAAT has catalytic activity, and it can catalyze L-glutamic acid and oxaloacetic acid to generate L-aspartic acid and α-ketoglutarate. Similarly, the reverse reaction solution was also subjected to HPLC detection. The results showed that the glutamic acid was detected in the products of the experimental group, but not in the control group (Fig. 4b). The HPLC analysis shows that the recombinant NhAAT is a bidirectional enzyme that catalyzes reversible reactions.

### Kinetic experiments

**Fig. 5:**
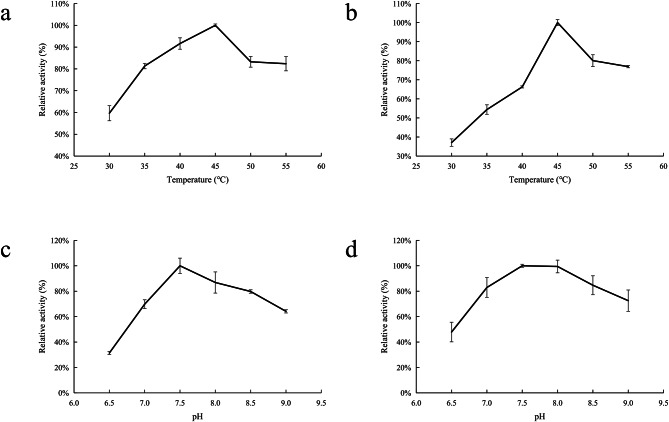
Influence of temperature and pH on the forward and reverse enzyme reaction of NhAAT. (a) Influence of temperature (30–55℃) on the forward activity of NhAAT; (b) Influence of temperature (30–55℃) on the reverse activity of NhAAT; (c) Influence of pH (6.5-9.0) on the forward activity of NhAAT; (d) Influence of pH (6.5-9.0) on the reverse activity of NhAAT. The reaction was carried out under corresponding conditions for 3 min. All values represent the mean ± SD calculated from three assays

**Fig. 6:**
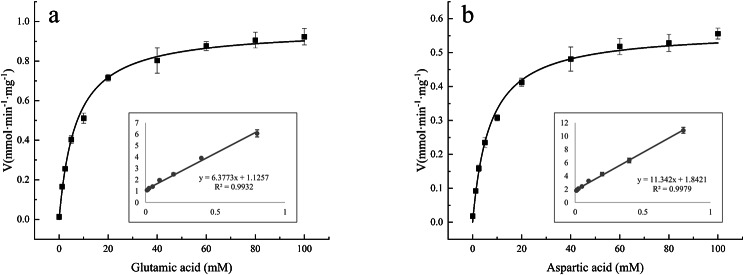
Kinetic analysis of NhAAT. (a) The *K*_*m*_ values of NhAAT for L-glutamic acid; (b) The *K*_*m*_ values of NhAAT for L-aspartic acid. All values represent the mean ± SD calculated from three assays

The enzyme activity was determined by measuring the variation in absorbance at 340 nm. The results of enzymatic characterization illustrated that the NhAAT has the highest catalytic activity at 45 °C and pH 7.5 in both forward and reverse reactions (Fig. 5). The calculated *K*_*m*_ values of NhAAT was 5.67 and 6.16 mM for L-glutamic acid and L-aspartic acid, respectively (Fig. 6).

### Quantitative real-time PCR analysis

**Fig. 7:**
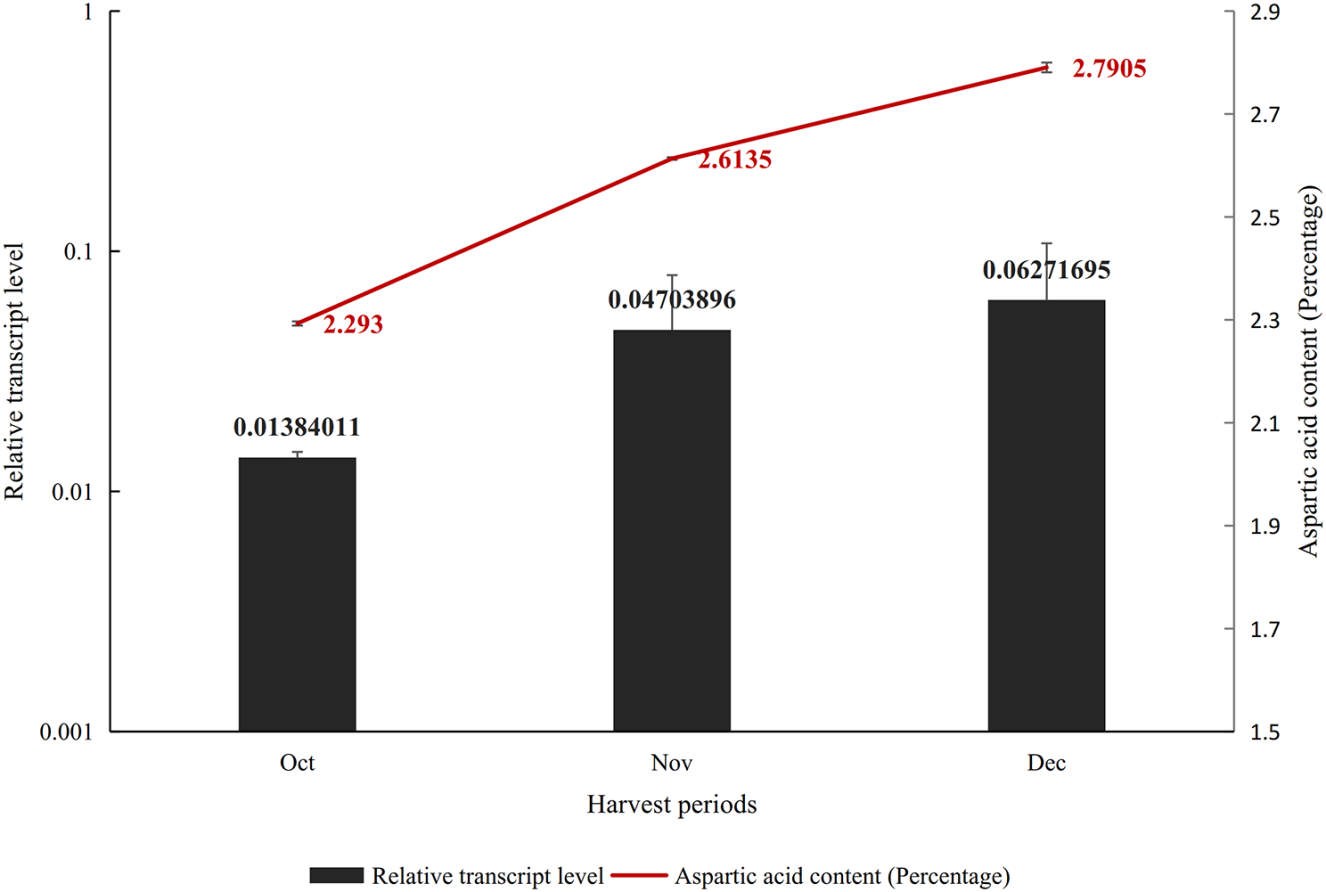
Transcription profiles of *NhAAT* and aspartic acid content in *N. haitanensis* sampled from different harvest periods. “percentage” means the weight ratio of amino acids to the dry weight of *N. haitanensis*. “Relative transcription level” means ratio or fold change between the amount of target gene in the experimental sample and that in the control sample. The measurement of aspartic acid content was conducted with two replicates, and the RT-PCR was conducted with three replicates

The trend of aspartic acid content in *N. haitanensis* was consistent with that of *NhAAT* expression level (Fig. 7). Quantitative analysis showed that the *NhAAT* expression of *N. haitanensis* collected in late harvest (Dec) was 4.5 times that of *N. haitanensis* collected in early harvest (Oct), while the aspartic acid content of *N. haitanensis* collected in late harvest (Dec) was 1.2 times that of *N. haitanensis* collected in early harvest (Oct). The change trend of both is consistent, indicating that AAT may play a role in aspartic acid content.

## Discussion

AATs play a key role in aspartic acid catabolism and biosynthesis as well as in the metabolic regulation of carbon and nitrogen metabolism [9]. In this study, through the analysis of *N. haitanensis* transcriptome, we isolated an *NhAAT* sequence.

The *NhAAT* encodes 429 amino acids, similar in molecular weight to AATs in other species. It contains the Aminotran_1_2 domain and belongs to the transaminase superfamily. The X-ray crystallographic studies of AAT have been performed in *E. coli* [14], chicken [15], pig [16], *Thermotoga maritima* [17], *Schizosaccharomyces pombe* [18] and other species. The recent reports on the crystal structures of AATs show that the three-dimensional structures of aspartate aminotransferases are well conserved in various species. The three-dimensional structure prediction of NhAAT shows that its similarity with the AAT H143L mutant from *Sus scrofa* (porcine) is 47.69%. And like other AATs, NhAAT is predicted to be a homo-dimer structure composed of two identical subunits. AAT is a typical PLP-dependent enzyme, and PLP is a basic, multifunctional enzymatic cofactor for catalyzing various chemical reactions involving amino acid metabolism [19]. The conserved protein domains of NhAAT were predicted by using NCBI Conserved Domains analysis. Ten and eight amino acid residues were predicted to be responsible for PLP binding and dimerization, respectively. Some sites were conserved in AATs, and Ser^117^, Gly^118^, Thr^119^, Arg^275^, Asn^203^, Tyr^234^, Lys^267^ are found consistent with previous reports by sequence alignment analysis [18].

The HPLC method was initially used for the detection of enzymatic activity, which confirmed that NhAAT has activity to catalyze the transformation between aspartic acid and glutamic acid. This method does not require additional coupling enzyme, but the measurement time is long and requires a large amount of sample, which is not conducive to the subsequent determination of enzyme kinetics. At present, the commonly used detection methods of AAT activity mainly include Reitman Frankel and enzyme-linked method. The Reitman Frankel is generally used in clinical medical detection; while the enzyme-linked method is to detect enzyme activity by coupling glutamate dehydrogenase or malate dehydrogenase [10, 20]. Therefore, the enzyme-linked method by coupling with malate dehydrogenase or glutamate dehydrogenase was selected for the subsequent determination of optimal conditions and enzyme kinetic parameters.

In this study, the optimum temperature of NhAAT was 45℃, which was higher than the optimum growth temperature of *N. haitanensis*. In many reports, AATs are generally thermally stable, and thermal stability appears to be related to the amino acid composition [10]. The AAT of *Thermus thermophilus* HB8 has a higher proline (Pro) content (6.5%), which makes the enzyme rigid and thermotolerant [10, 21]. Some studies showed that Pro residue could contribute to protein stabilization to different degrees [21]. The same features are also found in the AAT from *Phormidium lapdideum* [22]. Here, we found that the Pro content of NhAAT is 6.1%, which may be the reason why the NhAAT has a higher optimum temperature of 45℃. The optimum temperature of AAT from *P. lapdideum* is 35 °C higher than the optimum growth temperature of *P. lapdideum* [22]. This shows that the optimal reaction temperature of AAT is not necessarily close to the optimal growth temperature of organisms.

We also measured the enzyme activity at different pH values. The result showed that the optimum pH of NhAAT was 7.5, which is close to the environmental habitat of *N. haitanensis*. Furthermore, NhAAT showed relatively high activity over a wide alkaline pH range. Previous reports showed that aspartate aminotransferase (AspAT) from *Bacillus circulans* contains an additional *N*-terminal 32 amino acid residues that forms two α-helix segments, establishing a continuous network of interactions on the molecular surface, which may be the reason why the enzyme adapts to alkaline conditions [10, 23]. However, the NhAAT in this study has a low similarity to the AspAT, and it does not have the two α-helix segments. Besides, the alkali resistance of the enzyme was also reported to be related with the composition of amino acids. Studies have shown that the adaptation to high alkalinity will be accompanied by an increase in the number of arginine and neutral hydrophilic amino acid residues and also by a decrease in lysine and negatively charged amino acids [24, 25]. In this study, the NhAAT contained a percentage of Lys (3.5%), Glu (3.3%), His(1.9%). The percentage of acidic amino acid residues (Glu) and Lys residues of NhAAT was less than that of the AAT from *G. thermopakistaniensis* [26], while the percentage of neutral hydrophilic amino acid residues (His) was more. What’s more, the optimum pH of AAT_Gt_ was 7.0, which is lower than that of NhAAT [26]. Therefore, the composition of amino acids may be the reason why the NhAAT has high activity over a wide alkaline pH range. But the specific mechanism of the high activity (> 60%) under alkaline conditions (pH = 9.0) of NhAAT needs to be further explored.

The calculated *K*_*m*_ values of NhAAT was 5.67 and 6.16 mM for L-glutamic acid and L-aspartic acid, respectively. The *K*_*m*_ values of NhAAT for L-glutamic acid was close to that of cyanobacteria *Phormidium lapideum* (5.7 mM) [22], and is lower than that of alfalfa *Medcago sativa* L. (18.5 mM) [27]. The *K*_*m*_ values of NhAAT for L-aspartic acid was close to that of *Bacillus subtilis* B3 (6.68 mM) [10] and *Trypanosoma brucei* (6.8 mM) [28]. The *K*_*m*_ values for L-glutamic acid are lower than that for L-aspartic acid, and the *V*_*m*_ values for L-glutamic acid are higher than that for L-aspartic acid. This result showed that NhAAT present much higher affinity for L-glutamic acid. Therefore, we conjected that NhAAT preferred to participate in the aspartic acid biosynthetic pathway compared with the glutamic acid biosynthesis. The biosynthesis of glutamic acid can be carried out through three main enzymatic pathways: glutamate dehydrogenase, glutamate synthase and transaminase [29]. Considering the final step of aspartic acid biosynthesis can only be catalyzed by AAT, we deduced that AAT plays a more critical role in aspartic acid biosynthesis than glutamic acid biosynthesis. Except for the in vitro activity measurement, we further measured the content of aspartic acid in *N. haitanensis* sampled from three different harvest periods. The result showed that the aspartic acid content was increase in Dec as compared to Oct. Wei et al. [30] also found that harvest time was an important factor influencing the nutrient composition of *N. haitanensis*. And the levels of aspartic acid contained in seaweeds were significantly increased over the harvest time. The positive correlation of aspartic acid content and the transcription of *NhAAT* indicated that NhAAT was important for aspartic acid biosynthesis.

## Materials and methods

### Sample collection

The *N. haitanensis* sample was collected from Putian, Fujian in October, November and December, 2019, respectively. The sample was washed three times with sterile ddH_2_O, and the surface moisture was absorbed with gauze. Then the sample was frozen in liquid nitrogen and stored at -80℃. The laver from the three different harvest periods were sent to Analysis & Detection Center, Institute of Oceanology, Chinese Academy of Sciences (IOCAS) for aspartic acid content measurement.

### Cloning and sequence analysis of NhAAT gene

Total RNA of *N. haitanensis* was extracted with the Plant RNA Kit (OMEGA, China), and then the RNA was converted into cDNA according to Transcriptor First Strand cDNA Synthesis Kit (Takara, Japan). The candidate NhAAT gene was retrieved from the transcriptome database of *N. haitanensis* (NCBI accession: PRJNA428906). The open reading frames (ORFs) of *NhAAT* was amplified with primer F (5’-CCGGAATTCATGACGACCACGGCGATGGCGCCGG-3’) and primer R(5’-CCCAAGCTTTTACTCCTGTTTGACGACGGTTTCC-3’). A total of 20 µL PCR reaction mixture was used, with 1 µL of each primer, 10 µL of 2×Phanta Master Mix (Vazyme, China), 1 µL of cDNA, 7 µL of ddH_2_O. PCR program was as follows: 98 °C for 5 min; 35 cycles of 98 °C for 10 s, 55 °C for 15 s, and 72 °C for 90 s; and 72 °C for 10 min. Then the amplified product was cloned into the vector TOPO, and the entire cloned regions (TOPO-NhAAT) were confirmed by sequencing (Vazyme, China).

The obtained NhAAT coding sequence was translated into amino acid sequence with ORF Finder [31]. The sequence was then aligned with other AAT proteins by CLUSTALW (https://www.genome.jp/tools-bin/clustalw), and the results was illustrated by ESPript [32]. The physical and chemical parameters (molecular weight, isoelectric point) of NhAAT were predicted with ProtParam [33]. The conserved protein domains of NhAAT were predicted by using NCBI and the motifs of NhAAT were analyzed by the MOTIF tool (http://www.genome.jp/tools/motif/). The subcellular localization of NhAAT was predicted by TargetP v1.1, and the SignalP v4.1 Server (http://www.cbs.dtu.dk/services/SignalP-4.1/) was used to predict signal peptides [34]. The transmembrane helices were predicted with the TMHMM Server v2.0 (http://www.cbs.dtu.dk/services/TMHMM/). The tertiary structure of NhAAT was predicted by SWISS-MODEL [35]. Based on the AATs from 12 species released in GenBank, a phylogenetic tree was constructed by MEGA v7.0 using the Maximum likelihood algorithm, and 10,000 bootstrap replications were performed.

### Expression and purification of NhAAT

The plasmids TOPO-NhAAT and pCold-I were extracted according to the steps of TaKaRa MiniBEST Plasma Purification Kit (Takara, Japan). The extracted plasmids were digested with *Hin*dIII and *Eco*RI, and then were linked by T_4_ ligase, resulting in the fusion plasmid pCold-NhAAT. The *E. coli* strain BL21 plysS was transformed with pCold-NhAAT. The transformants were cultivated at 37℃ with shaking in Luria-Bertani (LB) medium containing 100 µg·mL^–1^ of ampicillin and 20 µg·mL^–1^ of chloramphenicol until OD_600_ reaching about 0.6. Flasks containing the cultures were supplemented with IPTG at a final concentration of 0.1 mM. After incubation at 15℃ for a further 24 h with vigorous shaking, the cells were harvested by centrifugation at 4,500 rpm and 4℃ for 30 min.

The cell pellets were resuspended in a buffer containing 20 mM sodium phosphate, 500 mM NaCl, 5% glycerol and 20 mM imidazole buffer at pH 8.0. Cells were lysed by sonication, and cell debris was removed by centrifugation at 12,000 rpm for 45 min. The overexpressed PhAAT was purified by chromatographic step using the ÄKTA Pure system (GE Healthcare, Fairfield, CA, USA) equipped with a His HP (GE Healthcare, Fairfield, UK). The column was equilibrated with 50 mL (10 column volumes) of buffer A (20 mM sodium phosphate, 20 mM imidazole, 500 mM NaCl, and 5% glycerin; pH 8.0) at a flow rate of 5 mL·min^–1^. Then 150 mL sample (bacterial extract diluted 5 times by buffer A) was injected at a rate of 1.2 mL·min^–1^. The non-adherent proteins were removed by rinsing with 20 volumes of buffer A. And then the protein was eluted by a gradient increase in the proportion of buffer B (20 mM sodium phosphate, 500 mM imidazole, 500 mM NaCl, and 5% glycerin; pH 8.0) at a rate of 3 mL·min^–1^. The eluted fractions were further purified using gel filtration with a Superdex 200 column (ÄKTA FPLC system; Amersham Pharmacia, Sweden), which equilibrated with buffer C (20 mM sodium phosphate, 150 mM NaCl, and 5% glycerin; pH 8.0). The elution was tested for the presence of the target protein by Western blot using Anti His-Tag mouse monoclonal antibody and Goat anti-mouse IgG (HRP conjugated) (CWBIO, Beijing, China), after separated by 12% sodium dodecyl sulphate-polyacrylamide gel electrophoresis (SDS-PAGE). The concentration of protein was determined with the BCA Protein Assay Kit (Vazyme, Nanjing, China).

### Determination of enzyme activities

NhAAT can catalyze the reversible reaction of L-glutamic acid and oxaloacetic acid to L-aspartic acid and α-ketoglutarate, and we measured the catalytic activity of these two directions respectively. The NhAAT purified by Ni^2+^-affinity chromatography was used to measure the catalytic activity. In the forward reaction (L-glutamate:oxaloacetate), the assay mixture contained 0.2 M sodium phosphate buffer (pH 8.5), 25 mM L-glutamic acid, 10 mM oxaloacetic acid, 0.125 mM PLP and NhAAT. In the reverse reaction (L-aspartate:α-ketoglutarate), the assay mixture contained: 0.2 M sodium phosphate buffer (pH 8.5), 25 mM L-aspartic acid, 10 mM α-ketoglutarate, 0.125 mM PLP and NhAAT. The reaction solution was sent to IOCAS for amino acid content detection.

### Kinetic experiments

For determination of the optimal reaction conditions and kinetic parameters, an assay was established by coupling with malate dehydrogenase or glutamate dehydrogenase [10, 20]. The NhAAT purified by Superdex 200 column was used to measure the catalytic activity. In the forward reaction, the assay mixture contained 0.1 M sodium phosphate buffer, 25 mM L-glutamic acid, 20 mM oxaloacetic acid, 20 mM NH_4_Cl, 2 mM NADH, 5 U of glutamate dehydrogenase, 50 µM PLP and NhAAT. In the reverse reaction, the assay mixture contained 0.1 M sodium phosphate buffer, 25 mM L-aspartic acid, 20 mM α-ketoglutarate, 2 mM NADH, 0.5 U of malate dehydrogenase, 50 µM PLP and NhAAT. The reaction was monitored by the decrease of the absorbance of NADH at 340 nm.

To obtain the optimal reaction conditions for the NhAAT, we measured the enzyme activity at various temperatures (30, 35, 40, 45, 50, and 55℃) and different pH values (6.5, 7.0, 7.5, 8.0, 8.5, and 9.0). When the optimal reaction conditions for the NhAAT were determined, the reaction rate was measured at different concentrations of L-glutamic acid (0-100 mM) and L-aspartic acid (0-100 mM). The *K*_*m*_ and *V*_*m*_ values were calculated by the double reciprocal plot method [36].

### Quantitative real-time PCR analysis

The primers (Forward: 5’-CTATGCCGAGTGGAAGAAGG-3’; Reverse: 5’-GGAGAACATGCCAATCTGGT-3’) were selected for qRT-PCR analysis and the *EF*_*2*_ was used as an internal control [37]. The qRT-PCR was performed with TB Green qPCR Mix (Takara, Otsu, Japan) on a TP800 Thermal Cycler Dice (Takara, Otsu, Japan). The protocol was 95℃ for 30 s; 40 cycles of 95℃ for 5 s, 51℃ for 30 s, and 72℃ for 30 s.

## Electronic supplementary material

Below is the link to the electronic supplementary material.

Supplementary Material 1

## Data Availability

The datasets used in the current study are available from the transcriptome data of *N. haitanensis* (NCBI accession: PRJNA428906).

## Declarations

### Sample collection permission

The collection of experimental material has been approved by Pinghai Harbor Aquaculture Co. Ltd., Putian, Fujian province, China.
