# Supplementary material for: Isolation and functional verification of an aspartate aminotransferase gene from Neoporphyra haitanensis
Source: BMC Plant Biol. 2023 Mar 21;23:150. doi: 10.1186/s12870-023-04158-2 (PMC10029208; doi:10.1186/s12870-023-04158-2)
Supplement: Supplementary file 1 — Supplementary Material 1 [file 12870_2023_4158_MOESM1_ESM.pdf]

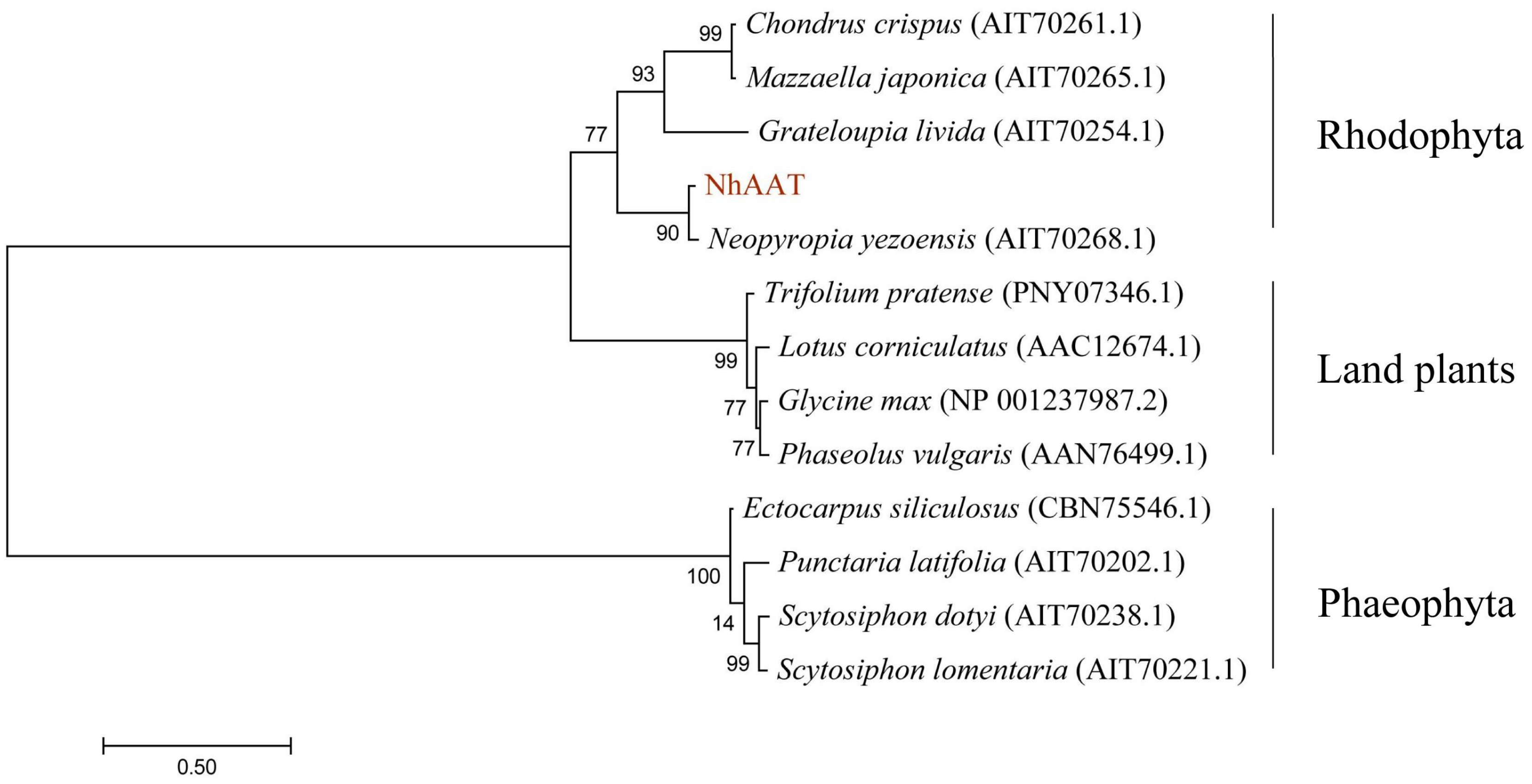

Figure S1. Phylogenetic tree constructed based on AAT protein sequences. The tree was constructed using the Maximum likelihood algorithm with 10,000 bootstrap replicates.

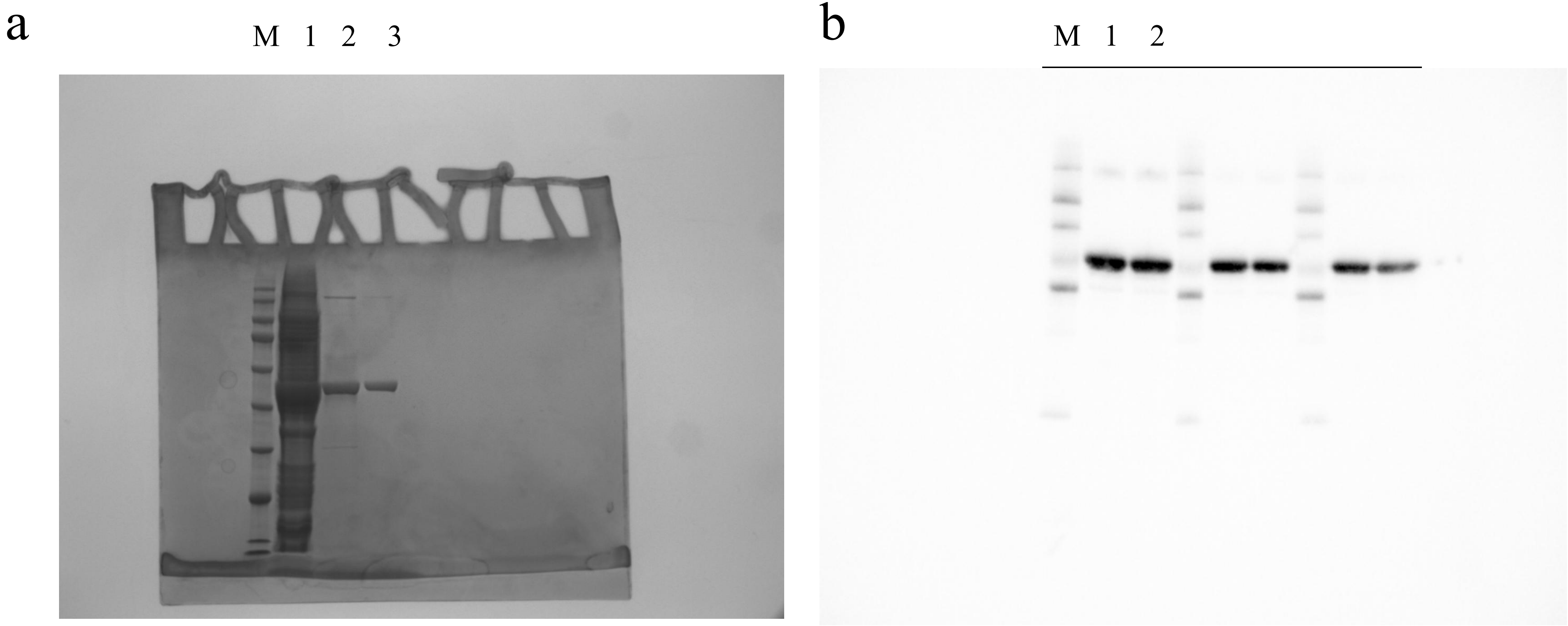

Figure S2. Original SDS-PAGE and Western blot results of recombinant NhAAT.
(a) SDS-PAGE analysis of recombinant NhAAT, M: protein ladder, Lane 1: crude enzyme; Lane 2: purified NhAAT after affinity purification, Lane 3: purified NhAAT after gel filtration purification.
(b) Western blot analysis of recombinant NhAAT, M: protein ladder, Lanes 1-2: purified NhAAT after gel filtration purification.
